# Functional analyses of the versicolorin B synthase gene in *Aspergillus flavus*

**DOI:** 10.1002/mbo3.471

**Published:** 2017-06-13

**Authors:** Silin Ren, Yuewei Yue, Yu Li, Xiaodong Guo, Shihua Wang

**Affiliations:** ^1^ Key Laboratory of Pathogenic Fungi and Mycotoxins of Fujian Province Key Laboratory of Biopesticide and Chemical Biology of Education Ministry, and School of Life Sciences Fujian Agriculture and Forestry University Fuzhou China

**Keywords:** aflatoxin, *Aspergillus flavus*, gene complementation strain, gene deletion strain, sclerotia

## Abstract

Aflatoxin is a toxic, carcinogenic mycotoxin primarily produced by *Aspergillus parasiticus* and *Aspergillus flavus*. Previous studies have predicted the existence of more than 20 genes in the gene cluster involved in aflatoxin biosynthesis. Among these genes, *aflK* encodes versicolorin B synthase, which converts versiconal to versicolorin B. Past research has investigated *aflK* in *A. parasiticus*, but few studies have characterized *aflK* in the animal, plant, and human pathogen *A. flavus*. To understand the potential role of *aflK* in *A. flavus*, its function was investigated here for the first time using gene replacement and gene complementation strategies. The *aflK* deletion‐mutant *ΔaflK* exhibited a significant decrease in sclerotial production and aflatoxin biosynthesis compared with wild‐type and the complementation strain *ΔaflK::aflK*. *ΔaflK* did not affect the ability of *A. flavus* to infect seeds, but downregulated aflatoxin production after seed infection. This is the first report of a relationship between *aflK* and sclerotial production in *A. flavus*, and our findings indicate that *aflK* regulates aflatoxin formation.

## 1 Introduction

The genus *Aspergillus* is a family of filamentous fungi with worldwide distribution, which is well‐studied for its important secondary metabolites, both beneficial and harmful. Within this genus, *Aspergillus flavus* infects a wide range of plants, animals, and humans as a disease‐causing pathogen (Amaike & Keller, 2011; Shieh et al., 1997). It can cause serious agricultural problems by contaminating important crops and producing extremely carcinogenic and highly toxic secondary metabolites known as aflatoxins (Amaike & Keller, 2011). Crops contaminated with aflatoxins pose a serious or even fatal threat to animals and humans. The gene cluster for aflatoxin biosynthesis in both *A. flavus* and *Aspergillus parasiticus* has been reported to encode at least 27 enzymes and regulatory factors within a 70 kb region (Yabe & Nakajima, 2004).

The functions of many genes within this cluster have been previously explored. For example, *aflX* is required for the conversion of versicolorin A, similar to the function of *aflN*,* aflM*, and *aflY* (Cary, Ehrlich, Bland, & Montalbano, 2006). The expression of *aflR* and *aflQ* of the aflatoxins biosynthesis cluster was analyzed Sweeney, Pamies, & Dobson (2000) using reverse transcription PCR, while Mayer, Bagnara, Farber, & Geisen (2003) examined the expression of *aflD* and the aflatoxin yield in wheat. Recently, Al‐Saad, Al‐Badran, Al‐Jumayli, Magan, & Rodriguez (2016) reported the impact of bacterial biocontrol agents on *aflD* and *aflR* expression and aflatoxin B_1_ production when *A. flavus* was grown under different environmental conditions and with various nutritional media. The expression of secondary metabolite biosynthesis cluster genes in *A. flavus*,* A. parasiticus*, and *Aspergillus oryzae* has also been compared (Ehrlich & Mack, 2014). Furthermore, aflatoxin biosynthesis gene expression was determined relative to water activity and temperature (Schmidt‐Heydt, Abdel‐Hadi, Magan, & Geisen, 2009), and we previously conducted a transcriptome analysis of *A. flavus* in response to water activity (Zhang et al., 2014).

Of the aflatoxin biosynthesis genes, *vbs*, also known as *aflK*, codes versicolorin B synthase (VBS) which is responsible for the conversion of versiconal to versicolorin B (Yu, Bhatnagar, & Ehrlich, 2002). *vbs* has been heterologous expressed, purified, isolated, and characterized in *A. parasiticus* (McGuire, Silva, Casillas, & Townsend, 1996; Silva, Minto, Barry, Holland, & Townsend, 1996; Silva & Townsend, 1997), and the distribution and subcellular localization of VBS was shown to change with respect to culture time in *A. parasiticus* (Chiou et al., 2004). Linz, Wee, & Roze (2014) hypothesized that VBS transport is tightly regulated by the timing and synthesis level of versicolorin A.

Almost all studies on *aflK* were performed in *A. parasiticus*, while few have investigated *aflK* in *A. flavus*. Therefore, to understand the potential role of *aflK* in *A. flavus*, we constructed the first known *aflK* deletion mutant and its complementation strain of *A. flavus*. We then analyzed the function of *aflK*, including its effect on growth, the formation of conidia and sclerotia, aflatoxin biosynthesis, and host colonization of *A. flavus*.

## 2 Materials and Methods

### 2.1 Phylogenetic tree and domain architecture

The protein sequence of AflK from *A. flavus* was identified and used in a BLAST search on the National Center for Biotechnology Information (NCBI) website (http://www.ncbi.nlm.nih.gov/). All available homogenous sequences from different organisms were downloaded and used to construct a phylogenetic tree with MEGA6.0 software and the neighbor‐joining method (Tamura, Stecher, Peterson, Filipski, & Kumar, 2013). Bootstrap analysis was set as 1,000 replicates. To visualize the AflK domain, information from the SMART database (http://smart.embl-heidelberg.de/) was subjected to DOG2.0 (Yang et al., 2016).

### 2.2 Fungal strains and media

*Aspergillus flavus* CA14PTS*ΔpyrG*, a uracil auxotrophic, was obtained from Prof. Chang P. K. (Chang, Scharfenstein, Wei, & Bhatnagar, 2010). *Aspergillus fumigatus* Af293 was a gift from Dr. Yang Liu (Institute of Agro‐Products Processing Science & Technology, Chinese Academy of Agricultural Sciences, Beijing, China). *Aspergillus * *flavus* was cultured on yeast extract sucrose (YES) agar plates for fungal growth, and YGTUU media was applied for the preparation of protoplasts. Yeast extract glucose (YGT) agar was used to screen gene deletion strains and to prepare protoplasts of deletion strains. Czapek's agar with pyrithiamine was used to screen complementation strains. Potato dextrose agar (PDA) was used for conidia production (Chang et al., 2011), and Wickerham media was used for sclerotial formation (Chang, Scharfenstein, Mack, & Ehrlich, 2012).

### 2.3 Protoplast preparation and fungal transformation

Protoplast preparation was performed as previously described (Chang et al., 2010; Gehret, Connelly, & Dumont, 2012; He, Price, OBrian, Georgianna, & Payne, 2007). Briefly, *A. flavus* CA14PTS*ΔpyrG* conidia were activated with YGTUU media and collected. After enzymolysis by β‐glucuronidase (Sigma, USA), lysing enzyme (Sigma, USA), and driselase (Sigma, USA), protoplasts were resuspended in 1.0 ml of STC buffer (10.0 mmol/L Tris‐HCl, pH 7.5, 10.0 mmol/L CaCl_2_, 1.2 mol/L sorbitol), then subpackaged (about 10^5^ protoplasts in 100 μl STC) and stored at −80°C. Deletion strain protoplasts were prepared using the same method.

Transformation was performed according to previously published techniques with minor modifications (Cary et al., 2006; He et al., 2007; Szewczyk et al., 2006). Protoplasts, the gel extraction product of fusion PCR or complementation vectors, and polyethylene glycol (PEG) buffer (50% PEG 4,000, 10.0 mmol/L NaH_2_PO_4_, pH 5.8, 10.0 mmol/L Tris‐HCl, pH 7.5, 50.0 mmol/L CaCl_2_, and 0.6 mmol/L KCl) were mixed with 10 mL of upper regeneration media (50.0 g/L of Czapek Solution Agar (BD Bioscience, NJ, USA), 1.0 mol/L sucrose, and 5.0 g/L agar), then plated on 10 ml of lower regeneration media (50.0 g/L of Czapek Solution Agar (BD Bioscience), 1.0 mol/L sucrose, and 15.0 g/L agar). The plates were cultured at 37°C for about 3 days.

### 2.4 Construction of deletion and complementation mutants

A previous publication was used as a reference to construct the *aflK* deletion strain *ΔaflK* and complementation strain *ΔaflK::aflK* (Ren et al., 2016). All gene sequences, including the open reading frame of *aflK*, the upstream and downstream sequence of *aflK*, and the *pyrG* sequence of *A. fumigatus* Af293, were searched for and downloaded from the NCBI website and *Aspergillus* Comparative Database (http://www.broadinstitute.org/annotation/genome/aspergillus_group/MultiHome.html). The PCR fusion program was performed as described by Szewczyk et al. (2006), and the PCR‐amplified complementary fragment was inserted into chromosomal integrating shuttle vector pPTR I (containing a pyrithiamine resistance gene as the selection marker, TAKARA, Japan) using *Kpn* I and *Hind* III (Thermo, USA) digestion and the ClonExpress II One Step Cloning Kit (C112‐01, Vazyme Biotech Co., Ltd, Nanjing, China). After bacterial transformation and PCR verification, the vectors were transformed into protoplasts of *ΔaflK* to construct the complementary strain *ΔaflK::aflK*. *pyrG* was amplified from *A. fumigatus* Af293 and transformed into *A. flavus* CA14PTS*ΔpyrG* protoplasts, which were then used as wild‐type (WT).

### 2.5 RNA extraction and reverse transcription (RT)‐PCR

The mycelia of WT, *ΔaflK*, and *ΔaflK::aflK* were harvested after cultured on YES agar for 48 hr. After grinding in liquid nitrogen, the result powder was suspended with TRIzol reagent (Biomarker Technologies, Beijing, China) for RNA extract. RNA molecules were purified with the DNA‐free kit (TransGen Biotech, Beijing, China) to remove genomic DNA. First‐strand cDNA was synthesized with the TransScript^®^ All‐in‐One First‐Strand cDNA Synthesis SuperMix.

### 2.6 Analysis of colony growth, conidial production, and sclerotial formation

To record the colony growth, 2 μl of 10^6^ spores/ml suspension of WT, *ΔaflK*, and *ΔaflK::aflK* were inoculated onto YES media. The diameters were measured until colonies filled the plate. For the enumeration of conidia, 2 μl spores suspension was inoculated onto PDA media, and cultured for 6 days at 37°C. Three pieces of 1‐cm diameter were harvested from the edge to the centre of each colony, and homogenized in 3 ml of distilled water. Then, spore number was counted manually with a hemocytometer. Conidiophores were observed according to a previous report (Yang et al., 2016). To analyze sclerotial production, 2 μl spores suspension of the three strains was cultured at 37°C for 10 days, then 75% ethanol was used to wash away conidia for the enumeration of sclerotia (Yang et al., 2016). The sclerotia were also observed microscopically. Each experiment was performed three times using four replicates. One‐way analysis of variance (ANOVA) was used for statistical testing.

### 2.7 Aflatoxin analysis

For aflatoxin biosynthesis analysis, 10^6^ spores of the three strains were incubated into YES liquid media, and cultured at 28°C for 6 days in the dark with shaking at 180 rpm. Aflatoxin was extracted with chloroform, then 10 ml of chloroform was transferred to a new tube and evaporated to dryness. Thin‐layer chromatography (TLC) was used to analyze aflatoxin production, and the outcome observed under ultraviolet light at 365 nm. The JD‐801 Computer‐aided Image Analysis System (JEDA Co., Nanjing, China) was used for the quantitative analysis of aflatoxin biosynthesis (Yang et al., 2016). Each experiment was performed three times using four replicates and analyzed by one‐way ANOVA. High‐performance liquid chromatography (HPLC) was used to confirm the presence of aflatoxin. The samples were filtered with 0.22 mm organic filtration, then analyzed by HPLC (Breeze^™^ HPLC, Waters, USA) on a MYCOTOX^™^ C_18_ column (NO. 1612124, 250 × 4.6 mm, Pickering Laboratories, USA) at 42°C. The running solvent was water:methanol:acetonitrile (56:22:22). A total of 20 μl of all samples was injected and run isocratically for 15 min at a flow rate of 1.0 ml/min. Aflatoxin was detected by a fluorescent detector with an emission wavelength of 455 nm and an excitation wavelength of 365 nm (Yang et al., 2016).

### 2.8 Seed infection

The ability of the three strains to infect peanut and maize was determined as described previously (Tsitsigiannis & Keller, 2006). After asepsis with 0.05% sodium hypochlorite and 75% ethanol, peanut cotyledons and maize kernels were inoculated with 10^5^ spores/ml suspension for 30 min, with shaking at 80 rpm. Then 20 cotyledons and 10 maize kernels were placed in culture dishes lined with three pieces of moist sterile filter paper to maintain humidity. A blank control was included in which the cotyledons within sterile water. Peanuts and maize were incubated at 28°C for 4 days in the dark, and humidity was maintained. Peanut seeds and maize kernels were then harvested in 50 ml tubes, and vortexed to release the spores into 15 ml of 0.05% Tween 20 (v/v in water); conidia were counted hemocytometrically. An equal amount of chloroform was used to extract aflatoxins. Each experiment was performed three times using four replicates and analyzed by one‐way ANOVA.

### 2.9 Formula of media

Yeast extract sucrose (YES) plate: 20.0 g/L yeast extract, 1.0 g/L MgSO_4_•7H_2_O, 150.0 g/L sucrose, and 15.0 g/L agar (Abdel‐Hadi, Schmidt‐Heydt, Parra, Geisen, & Magan, 2012; Calvo, Bok, Brooks, & Keller, 2004). Yeast extract glucose (YGT) agar: 5.0 g/L yeast extract, 20.0 g/L glucose, 1 ml of trace element solution per liter of media, and 15.0 g/L agar (Yang et al., 2016). YGTUU plate: 5.0 g/L yeast extract, 20.0 g/L glucose, 1 ml of trace element solution per liter of media, 1.0 mg/ml uracil, 1.0 mg/ml uridine, and 15.0 g/L agar (Yang et al., 2016). Czapek's agar: 50.0 g/L of Czapek solution agar (BD Bioscience, NJ, USA). Potato dextrose agar (PDA): 39.0 g/L of potato dextrose agar (BD Bioscience, NJ, USA). Wickerham media: 30.0 g/L sucrose, 3.0 g/L peptone, 5.0 g/L corn steep liquid, 2.0 g/L yeast extract, 2.0 g/L NaNO_3_, 0.1 g/L FeSO_4_ •7H_2_O, 1.0 g/L K_2_HPO_4_•H_2_O, 2.0 g/L dextrose, 0.2 g/L KCl, 0.5 g/L MgSO_4_•7H_2_O, pH 5.5 (Chang et al., 2012).

## 3 Results

### 3.1 Phylogenetic analysis and domain prediction of AflK in *A. flavus*

**Figure 1:**
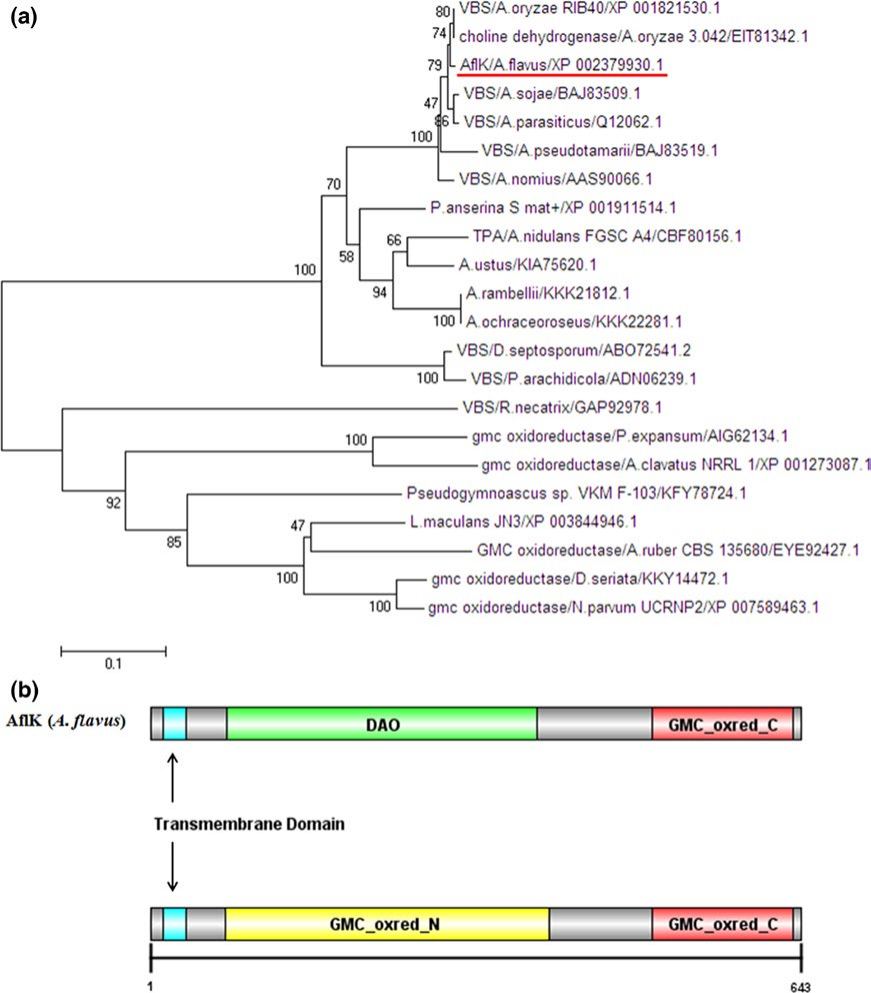
Phylogenetic tree and domain analysis of AflK in *A. flavus*. (a) Phylogenetic tree of AflK (versicolorin B synthase). AflK of *A. flavus* is marked with a red line. (b) Domain analysis of AflK

Protein sequences of AflK (versicolorin B synthase) from *A. flavus* and 21 homologous proteins were used to construct a phylogenetic tree. The protein sequences chosen as analogous to *A. flavus* AflK (XP_002379930.1) for the analysis were: versicolorin B synthase in *A. oryzae* RIB40 (XP_001821530.1), choline dehydrogenase in *A. oryzae* 3.042 (EIT81342.1), versicolorin B synthase in *Aspergillus nomius* (AAS90066.1), conserved hypothetical protein in *Aspergillus nidulans* FGSC A4 (CBF80156.1), hypothetical protein ARAM_004338 in *Aspergillus rambellii* (KKK21812.1), hypothetical protein AOCH_000141 in *Aspergillus ochraceoroseus* (KKK22281.1), versicolorin B synthase in *Dothistroma septosporum* (ABO72541.2), versicolorin B synthase in *Passalora arachidicola* (ADN06239.1), hypothetical protein in *Podospora anserina* S mat+ (XP_001911514.1), hypothetical protein HK57_00552 in *Aspergillus ustus* (KIA75620.1), versicolorin B synthase in *Aspergillus sojae* (BAJ83509.1), versicolorin B synthase in *A. parasiticus* (Q12062.1), a protein similar to glucose‐methanol‐choline (GMC) oxidoreductase in *Leptosphaeria maculans* JN3 (XP_003844946.1), putative versicolorin B synthase in *Rosellinia necatrix* (GAP92978.1), GMC oxidoreductase in *Penicillium expansum* (AIG62134.1), putative GMC oxidoreductase in *Diplodia seriata* (KKY14472.1), putative GMC oxidoreductase protein in *Neofusicoccum parvum* UCRNP2 (XP_007589463.1), hypothetical protein V499_02164 in *Pseudogymnoascus* sp. VKM F‐103 (KFY78724.1), GMC oxidoreductase in *Aspergillus clavatus* NRRL 1 (XP_001273087.1), GMC oxidoreductase‐like protein in *Aspergillus ruber* CBS 135680 (EYE92427.1), and versicolorin B synthase in *Aspergillus pseudotamarii* (BAJ83519.1). Among these protein sequences, the consistency between versicolorin B synthase in *A. oryzae* RIB40 (XP_001821530.1) and AflK in *A. flavus* (XP_002379930.1) was 99.69%, between choline dehydrogenase in *A. oryzae* 3.042 (EIT81342.1) and XP_002379930.1 was 99.53%, between versicolorin B synthase in *A. parasiticus* (Q12062.1) and XP_002379930.1 was 98.76%, and between conserved hypothetical protein in *A. nidulans* FGSC A4 (CBF80156.1) and XP_002379930.1 was 75.70%. As shown in Figure 1a, the phylogenetic tree demonstrated that AflK from *A. flavus* and VBS from other *Aspergillus* species belonged to the same clade. Within this clade, *A. flavus* AflK and *A*. oryzae VBS belonged to the same grade, VBS from *A. parasiticus* and *A. sojae* were in the same grade, but *A. flavus* AflK and *A. parasiticus* VBS were not. Based on the results of SMART, three domains were identified in AflK of *A. flavus*, including the transmembrane domain (from 13th to 35th amino acid), the C‐terminal of GMC oxidoreductase (GMC_oxred_C, from 496th to 635th amino acid) and D‐amino acid oxidase (DAO) domain (from 76th to 382nd amino acid) or the N‐terminal of GMC oxidoreductase (GMC_oxred_N, from 75th to 394th amino acid) (Figure 1b). These results suggested that AflK was conserved but relatively unique.

### 3.2 Growth and conidia production of WT, *ΔaflK*, and *ΔaflK::aflK*

**Figure 2:**
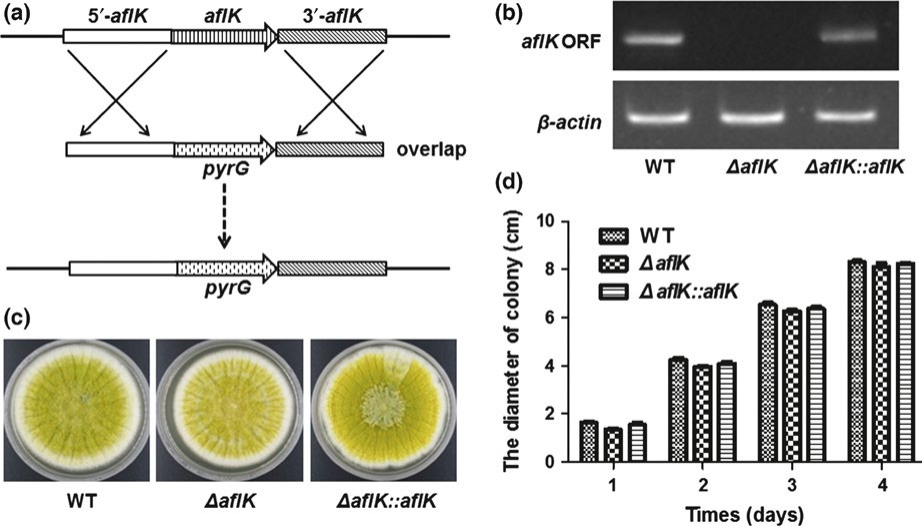
Construction of *∆aflK* and growth analysis of WT,* ∆aflK*, and *∆aflK::aflK*. (a) Schematic of the replacement of *aflK* (versicolorin B synthase gene) with *pyrG*. (b) cDNA verification of *∆aflK* and *∆aflK::aflK*. The *actin* gene was used as a reference. ORF, open reading frame of *aflK*. (c) Morphological phenotypes of the three strains cultivated with YES. (d) Quantification analysis of colony diameter

**Figure 3:**
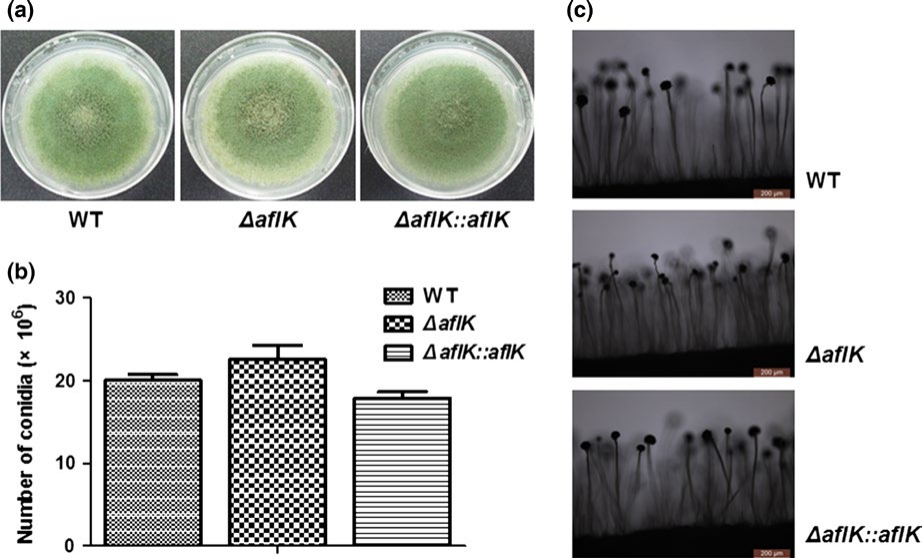
Conidiation phenotype of WT,* ∆aflK*, and *∆aflK::aflK*. (a) Morphological phenotypes of WT,* ∆aflK*, and *∆aflK::aflK* cultivated with PDA media. (b) Quantification analysis of conidia. (c) Microphotograph of conidiophore cultivated with YES media

*aflK* is one element of the aflatoxin biosynthesis cluster. Although it has been fully studied in *A. parasiticus*, our phylogenetic analysis showed that AflK of *A. flavus* and VBS of *A. parasiticus* were in different grades of one clade suggesting a possible difference or specialty of AflK in *A. flavus*. To study the potential function of *A. flavus aflK*, we constructed an *aflK* deletion mutant by replacing its open reading frame with a selection marker, *pyrG*. All primers used in this study are listed in Table 1, and a schematic of this method is given in Figure 2a. Deletion and complementation mutants of *aflK* were successfully obtained as verified by PCR of selected transformants (data not shown); the failure or restoration of gene expression in the Δ*aflK* mutant and *ΔaflK::aflK* strain was confirmed by RT‐PCR (Figure 2b**)**. Next, we studied the role of AflK in *A*. *flavus* development. As shown in Figures 2c and d, no significant differences were observed in the colonial phenotypes or diameters when WT, *ΔaflK*, and *ΔaflK::aflK* were cultured on YES plates. The effect of *aflK* on conidiation was also studied by culturing WT, *ΔaflK*, and *ΔaflK::aflK* onto PDA plates and YES agar at 37°C. No significant difference was found in the conidia production of the three strains (Figure 3a and Figure 3b**)**. Figure 3c shows that the conidiophores of these strains cultured on YES agar were identical. These results indicated that *aflK* does not affect the growth and conidia production of *A. flavus*.

**Table 1:** Primers used in this study

Primers	Sequence(5′‐3′)	Application
*aflK‐*1	CGGAGCCTCGTTCCAAAT	To amplify upstream fragment of *aflK*
*aflK*‐3	ACTGACAATTTGAAGCTGGAATCCCTCAACGGGCTTTC	
*aflK*‐4	TCAGGACCACATACTCTACCATACCGTTCTCGAACTCGC	To amplify downstream fragment of *aflK*
*aflK*‐6	GTTGGTGGACGTTGATGG	
*aflK*‐2	CTCGTCCTTCCATCCTCT	To amplify overlap fragment to replace *aflK*
*aflK*‐5	CTGTTCGTCAAAGCCCTA	
*pyrG*‐F	GCCTCAAACAATGCTCTTCACCC	To amplify *pyrG*
*pyrG*‐R	GTCTGAGAGGAGGCACTGATGC	
P801‐R	CAGGAGTTCTCGGGTTGTCG	To verify overlap fragment of *aflK*
P1020‐F	ATCGGCAATACCGTCCAGAAGC	
*aflK*‐OF	CTCAGGGCTTCTCCAATG	To verify ORF of *aflK*
*aflK*‐OR	CTCGGGTCCAGCAACG	
*aflK*‐com‐F	CTATGACCATGATTACGCCAAGCTTAGGATCAGATCTGGGATG	To amplify complementation vector of *aflK*
*aflK*‐com‐R	CCAGTGAATTCGAGCTCGGTACCTGATAGTTTCATCGCC	
*actin‐*F	CAGCCGCTAAGAGTTCCAG	To amplify *actin*
*actin‐*R	CACCGATCCAAACCGAGTAC	

### 3.3 Reduction of aflatoxin biosynthesis in *ΔaflK*

**Figure 4:**
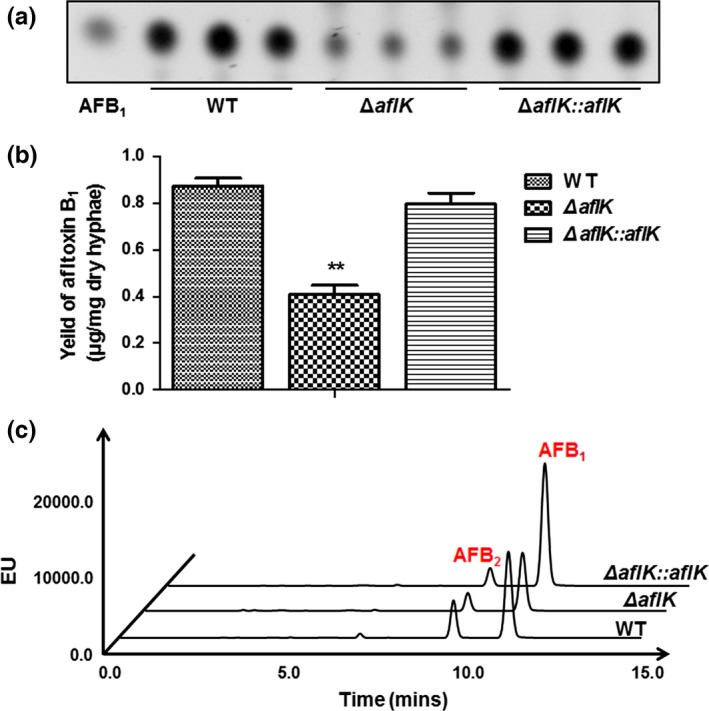
Aflatoxin production of WT,* ∆aflK*, and *∆aflK::aflK*. (a) TLC analysis of aflatoxin B_1_ (AFB_1_) production. (b) Quantification analysis of AFB_1_ as in (a). Asterisks ** represent a significant difference (*p*‐value < .01). (c) HPLC analysis of aflatoxin production. The peak of AFB_1_ is almost at 10.9 min and AFB_2_ is almost at 9.1 min

*aflK* is located in the aflatoxin synthesis gene cluster of *A. flavus*, so we next investigated aflatoxin biosynthesis after the deletion of *aflK*. To examine the role played by *aflK* in aflatoxin synthesis, we tested aflatoxin production in WT, *∆aflK*, and *∆aflK::aflK* by TLC at 6^th^ day. The aflatoxin B_1_ (AFB_1_) produced by *ΔaflK* was reduced compared with that of WT and *ΔaflK::aflK* (Figure 4a**)**, and Figure 4b shows the quantitative analysis of AFB_1_ production. HPLC was also used to confirm these results, which indicated that AFB_1_ production of *ΔaflK* was lower than that of WT and *ΔaflK::aflK* strains (Figure 4c). The original maps of TLC and HPLC are shown as the representative result. These findings suggested that *aflK* plays a vital role in aflatoxin biosynthesis in *A. flavus*.

### 3.4 Negative role of *aflK* in sclerotial production

**Figure 5:**
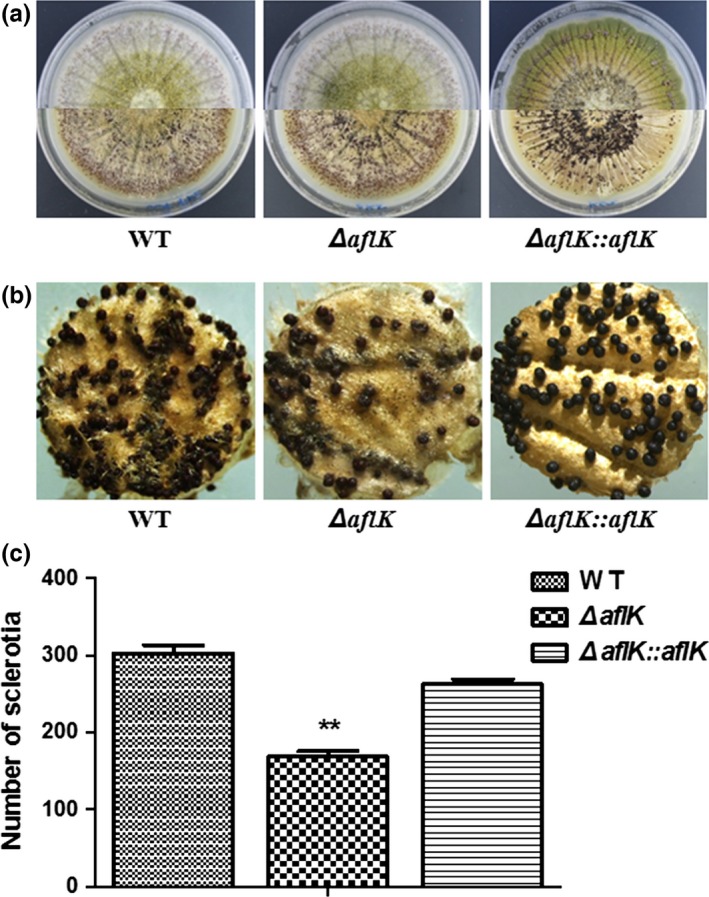
Sclerotial phenotype of WT,* ∆aflK*, and *∆aflK::aflK*. (a) Phenotypic characterization of WT,* ∆aflK*, and *∆aflK::aflK* before (upper section) and after (lower section) ethanol treatment. (b) Microphotograph of sclerotia. (c) Quantification analysis of sclerotia. Asterisks ** represent a significant difference (*p*‐value < .01)

Previous studies have shown that sclerotial production may be associated with aflatoxin biosynthesis (Calvo et al., 2004; Duran, Cary, & Calvo, 2007). Therefore, we determined the effect of *aflK* on sclerotial production by culturing the strains on Wickerham agar at 37°C for 7 days in the dark. The conidia were washed away with 75% ethanol to visualize the sclerotial phenotypes, as shown in Figure 5a and b. Surprisingly, the sclerotial production of *ΔaflK* was significantly decreased compared with that of WT and *ΔaflK::aflK* strains (Figure 5c), indicating that *aflK* plays an important role in the sclerotial production of *A. flavus*.

### 3.5 Seed infections of WT, *ΔaflK*, and *ΔaflK::aflK*

**Figure 6:**
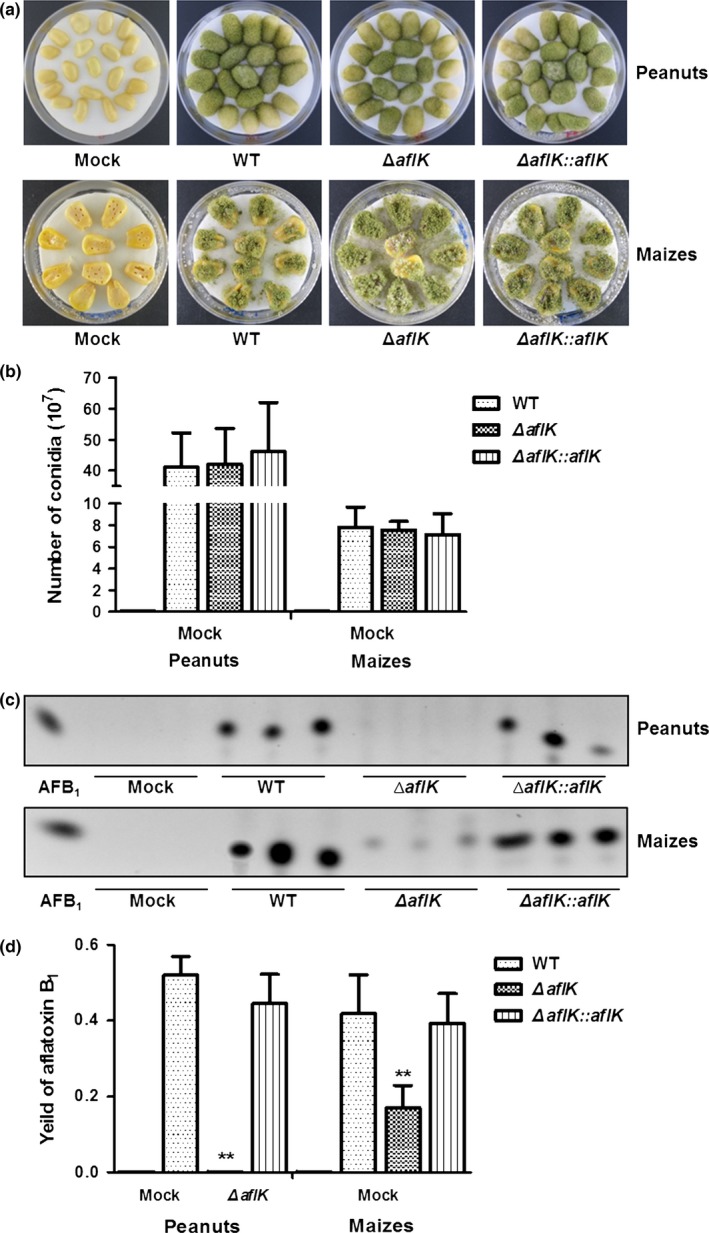
Seed infection ability and aflatoxin production (after infection) of WT,* ∆aflK*, and *∆aflK::aflK*. (a) Peanut cotyledons and maize kernels were infected by the three strains. (b) Quantification analysis of conidia production after infection. (c) Thin‐layer chromatography analysis of AFB_1_ production. (d) Quantification analysis of AFB_1_. Asterisks ** represent a significant difference (*p*‐value < .01)

*A. flavus* has a high number of diverse virulence factors and is a biological hazard to crops (Amaike & Keller, 2011). Based on the observed changes in aflatoxin production and sclerotial production caused by *aflK* deletion, we determined whether the *aflK* deletion also affected the ability of the strain to infect seeds. Peanut and maize were treated with conidia from WT, *ΔaflK*, and *ΔaflK::aflK* strains, but no obvious infection ability of *A. flavus* was observed (Figure 6a), and there was no significant difference in the number of conidia after infection of seeds by WT, *ΔaflK*, and *ΔaflK::aflK* (Figure 6b). Aflatoxin was extracted with chloroform from infected peanut cotyledons and maize kernels, showing that the deletion of *aflK* reduced AFB_1_ production after seed infection (Figure 6c). Quantification analysis of AFB_1_ was shown in Figure 6d, based on the results of Figure 6c. Together, these results illustrated that the deletion of *aflK* did not influence the infection ability of *A. flavus*, but reduced the level of aflatoxin biosynthesis after seed infection.

## 4 Discussion

In this study, we found that VBS was relative conserved in *Aspergillus* sp., and that it also shares a high level of identity with GMC oxidoreductase in other pathogenic microorganisms. The domain prediction analysis further confirmed this finding, revealing a probable DAO domain or GMC domain in the AflK protein. The DAO domain is the characteristic structure of D‐amino acid oxidase, which catalyses the oxidation of neutral and basic D‐amino acids (Miyano et al., 1991). D‐amino acid oxidase is located in peroxisomes (Pollegioni, Piubelli, Sacchi, Pilone, & Molla, 2007), in which the early steps of aflatoxin biosynthesis are likely to occur, and were shown by Chanda et al. (2009) to supply acetyl‐CoA for aflatoxin synthesis. This suggests that the *A. flavus* AflK protein plays a pivotal role in aflatoxin biosynthesis and is determined by its domain. However, the overall level of homology between *A. flavus* AflK and common *Aspergillus* sp. (*A*. *oryzae*,* A. parasiticus*, and *A. nidulans*) did not always exceed 99%. Phylogenetic tree analysis showed further differences between *A. flavus* AflK and its homologous proteins.

Previous investigations of the aflatoxin biosynthesis pathway fully researched its enzymes, substrates, and products, and largely focused on the detection and control of aflatoxin production (Bennett & Christensen, 1983; Bhatnagar, Ehrlich, & Cleveland, 2003; Biollaz, Buchi, & Milne, 1970; Cary, Szerszen, & Calvo, 2009; Ellis, Smith, Simpson, & Oldham, 1991; Gupta, Prasanna, Viswanathan, & Venkitasubramanian, 1975; Maggon, Gupta, & Venkitasubramanian, 1977). Thus, such studies have included the examination of *aflK*, encoding versicolorin B synthase (Yabe & Nakajima, 2004; Yu et al., 2002, 2004), revealing its heterologous expression, purification, isolation, characterization, distribution, and subcellular localization in *A. parasiticus* (Chiou et al., 2004; McGuire et al., 1996; Silva & Townsend, 1997; Silva et al., 1996). Because few *aflK* studies have been carried out in *A. flavus*, we performed homologous recombination to obtain an *aflK* deletion mutant and used a commercial chromosomal integrating shuttle vector to produce an *aflK* complementation strain. It is the first time that a gene knockout method is conducted for *A. flavus* ∆*aflK*. Our research focused not only on the role of *aflK* in aflatoxin biosynthesis, but also in development and infection of *A. flavus*.

Our results showed that the deletion of *aflK* had no effect on growth and conidiation of *A. flavus*. However, *aflK* appeared to play an important role in aflatoxin production, which is consistent with previous studies about aflatoxin biosynthesis (Yabe & Nakajima, 2004; Yu et al., 2002, 2004). Moreover, the *aflK* deletion did not completely inhibit the production of aflatoxin, as previously seen following the deletion of *A. parasiticus aflE* (Trail, Chang, Cary, & Linz, 1994). This may be because an alternative protein compensated for the role of AflK. A BLAST search of *A. flavus* AflK identified other proteins with different accession numbers in *A. flavus* homologous to VBS. Interestingly, we found that the inactivation of *aflK* decreased sclerotial production in *A. flavus*. The synchronous variation between sclerotia and aflatoxin was comparable with previous studies of *veA* and *laeA* (Amaike & Keller, 2009; Calvo et al., 2004; Duran et al., 2007; Kale et al., 2008). Aflatoxin biosynthesis in *A. flavus* and *A. parasiticus* was previously shown to be regulated by *veA* and *laeA*, which are also necessary for sclerotial formation (Calvo et al., 2004; Kale et al., 2008). We therefore speculate that sclerotial formation was decreased in response to the deletion of *aflK*, under the effects of these regulatory genes. But this is the first time to reveal the effect of aflatoxin biosynthesis gene *aflK* on sclerotial formation. Our results therefore indicate that *aflK* has an important role in aflatoxin biosynthesis and sclerotial production in *A. flavus*.

We found that an *aflK* deletion did not affect the infection of *A. flavus*, but caused a notable decrease in aflatoxin production after infection of peanut and maize. The *aflK* deletion also had no effect on conidia production after infection, consistent with our findings that *aflK* did not affect growth or conidia production, but regulated aflatoxin biosynthesis of *A. flavus*. This could reflect the fact that *A. flavus* virulence is a multifactorial process closely connected with secondary metabolism, developmental linkage of sporulation, and other aspects of *A. flavus* (Amaike & Keller, 2011). Hence, because *aflK* is only one part of the aflatoxin biosynthesis gene cluster, it did not influence the infection of *A. flavus*, but decreased aflatoxin production after infection.

In conclusion, AflK appears to be a conservative and unique oxidoreductase of *Aspergillus* sp. This is the first report of the influence of *aflK* on both sclerotial formation and aflatoxin biosynthesis in *A. flavus*. A more comprehensive investigation of the role of *aflK* in the development and pathogenicity of *A. flavus* will be conducted in future studies.

## Conflict of Interest

None declared.
